# Exaggerated Liver Injury Induced by Renal Ischemia Reperfusion in Diabetes: Effect of Exenatide

**DOI:** 10.4103/1319-3767.65187

**Published:** 2010-07

**Authors:** Jitendra D. Vaghasiya, Navin R. Sheth, Yagnik S. Bhalodia, Nurudin P. Jivani

**Affiliations:** Department of Pharmacology, Jodhpur National University, Jodhpur, India; 1 Department of Pharmaceutical Sciences, Saurashtra University, Rajkot, Gujarat, India

**Keywords:** Exenatide, diabetes type 2, ischemia reperfusion, liver injury, oxidative stress

## Abstract

**Background/Aim:**

This study was designed to investigate the possible effect of exenatide (Glucagon like Peptide-1 receptor agonist) on liver injury (distant organ) induced by renal ischemia reperfusion (IR) in diabetic rats.

**Materials and Methods:**

*In vivo* renal IR was performed in both type 2 diabetic and normal rats. Each protocol comprised ischemia for 30 minutes followed by reperfusion for 24 hours and a treatment period of 14 days before induction of ischemia.

**Results:**

Lipid peroxidation, xanthine oxidase activity, myeloperoxidase activity and nitric oxide level in liver tissue were significantly increased (*P* < 0.01, *P* < 0.001, *P* < 0.001, *P* < 0.05, respectively), after IR in diabetic rats compared to normal rats. Antioxidant enzymes like glutathione, superoxide dismutase, catalase and glutathione peroxidase were significantly reduced (*P* < 0.05, *P* < 0.05, *P* < 0.01, *P* < 0.05, respectively), after IR in diabetic rats compared to normal rats. Exenatide treatment significantly normalized (*P* < 0.01), these biochemical parameters in treated rats compared to diabetic IR rats. Serum creatinine phosphokinase activity and liver function enzymes were also significantly normalized (*P* < 0.001, *P* < 0.001, respectively), after administration of exenatide.

**Conclusion:**

Exenatide exerted protective effect on exaggerated remote organ (liver) injury induced by renal IR in diabetes.

Liver and kidney are important regulators of body homeostasis and are involved in excreting the toxic products of metabolism and exogenous drugs.[1] Any injury to either the renal or liver tissue, may affect the other. There is now growing evidence that some endocrine disorders (in particular, diabetes mellitus) may actually cause liver disease, but it has been very difficult to sort out cause and effect. Interestingly, although nonalcoholic steatohepatitis had initially been strongly associated with obesity and diabetes mellitus (DM), it later became apparent that these risk factors do not have to be present for nonalcoholic steatohepatitis to develop.[2,3] Rather, it may be that insulin resistance could be the common denominator underlying liver disease, although the mechanism by which this abnormality results in liver disease remains uncertain. DM causes organ dysfunctioning and increases the sensitivity of organs to damages. Diabetic patients may need renal transplantation in their later life due to diabetic nephropathy. The ischemia reperfusion (IR) injury is one of the dangerous complications of this procedure. The short period of ischemia (30 minutes) in diabetes has been demonstrated to cause reversible renal failure, leading to progressive injury with end-stage renal disease.[4] Various investigators have reported that renal IR causes distant-organ injury such as liver injury.[5,6]

Reactive oxygen species (ROS) and nitric oxide (NO) play an important role in mediating cell damage during IR injury.[7,8] Inflammation contributes substantially to the pathogenesis of IR with a central role for particular cells, adhesion molecules and cytokines.[9] Neutrophils are the inflammatory cells that abundantly produce ROS during IR injury. Myeloperoxidase (MPO) is found in neutrophils and catalyzes the formation of hypochlorous acid (HOCl), a toxic agent to cellular components, and initiates oxidative injury.[10] Renal IR causes tissue injury by oxygen radicals and oxidative stress, caused by an imbalance between production of ROS and antioxidant capacity.[11]

Liver injury is one of the distant-organ damages induced by kidney IR. Acute renal failure associated with liver disease is a commonly encountered clinical problem of varied etiology.[4] It is believed that IR injury induces inflammatory response, which elicits tissue damage in a number of organs in which reactive oxygen and nitrogen species play a key role in the pathophysiology of renal IR injury.[11,4] It has been demonstrated that renal IR injury might cause liver oxidative stress and increase lipid peroxidation in liver tissue.[12] It is well reported that the liver tissue of rat decreases antioxidant enzyme activities after renal IR.[5]

Exenatide, a synthetic version of Exe-4, is presently the only GLP-1 receptor agonist approved for clinical use by the Federal Drug Administration in treatment of type 2 diabetes. Exenatide exhibits insulinotropic and insulinomimetic properties via the G-protein–coupled GLP-1 receptor, which has been reported to be expressed in various tissues.[13,14] In view of the reported efficacy of GLP-1 in ischemia reperfusion injury,[15–17] the present study was designed to investigate the effect of exenatide on liver injury (distant organ) induced by renal IR in diabetic rats.

## MATERIALS AND METHODS

### Induction of diabetes type 2

Healthy adult Wistar rats (either sex) weighing 200 to 250 g were used. The experiment and protocol described in the present study were approved by the Institutional Animal Ethics Committee (IAEC) of Smt. R.B.P.M.C., Atkot, and the study was carried out with permission from the Committee for the Purpose of Control and Supervision of Experiments on Animals (CPCSEA), Ministry of Social Justice and Empowerment, Government of India. Diabetes type 2 in rats was induced by administration of nicotinamide (NAD) (230 mg/kg, *i.p*.), 15 minutes prior to the single dose of streptozotocin (STZ) (65 mg/kg, *i.v*.).[18] Control animals received an equal volume of saline. Food, water consumption, weight gain and blood glucose levels were recorded to monitor the degree of diabetes. A period of four weeks was maintained between induction of diabetes and ischemic injury.

### Induction of renal IR injury in diabetic rats

**Figure 1:**
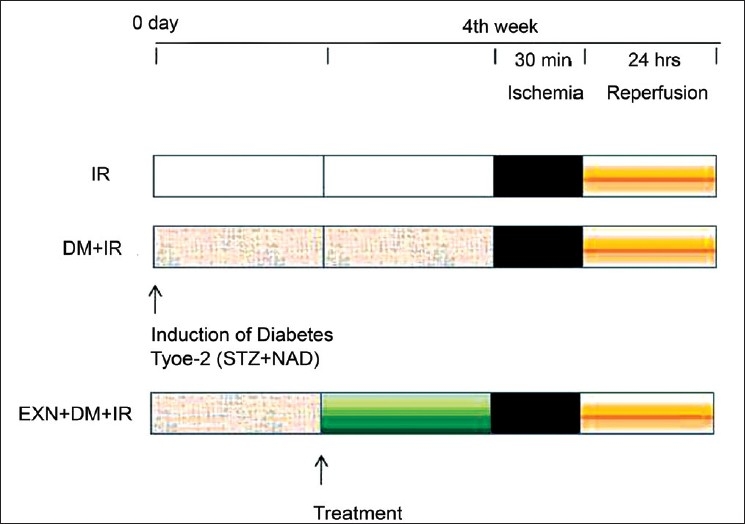
Scheme showing experimental protocols employed. Each protocol comprised of the following phases: ischemia, 30 minutes; reperfusion, 24 hours; treatment period, 14 days before induction of ischemia

Diabetic rats were anesthetized with ketamine (60 mg/kg, *i.p*.) and diazepam (5 mg/kg, *i.p*.). Body temperature was maintained throughout surgery at 37°C ± 0.5°C. The skin on the back was shaved and disinfected with povidone iodine solution. Surgical exposure of the left and right renal pedicles was obtained in all rats via midline incision. To induce renal ischemia, both renal pedicles were occluded for 30 minutes with vascular clamps. After 30 minutes of occlusion, the clamps were removed and kidneys observed while they underwent reperfusion for 24 hours. Rats were randomly divided into five different groups (n= 6) [Figure 1]: group 1- normal control, group 2- diabetic control, group 3- renal IR injury, group 4- diabetes + renal IR injury, group 5- diabetes + exenatide treatment + renal IR injury. In the treatment group, exenatide (Sigma, St. Louis, MO) was injected in a dose of 10 mcg, subcutaneous, twice a day for two weeks before the induction of IR. At the end of each *in vivo* study, rats were sacrificed and livers were quickly removed and placed into liquid nitrogen and then stored at –70°C until assayed for oxidant and antioxidant parameters.

### Estimation of liver function

Estimation of liver function was carried out by measuring marker enzymes of liver function, like aspartate aminotransferase (AST), alanine aminotransferase (ALT) and alkaline phosphatase (ALP), by using the kit manufactured by Span Diagnostic Ltd., India.

### Estimation of lipid peroxidation and antioxidant enzymes

Liver was removed and kept in cold conditions (precooled in Petri dish inverted on ice). It was cross-chopped with surgical scalpel into fine slices in chilled 0.25-M sucrose and quickly blotted on a filter paper. The tissue was minced and homogenized in 10-mM Tris-HCl buffer, pH 7.4 (10% w/v), with 25 strokes of tight Teflon pestle of glass homogenizer at a speed of 2500 rpm. The clear supernatant was used for assays of lipid peroxidation (MDA content) and endogenous antioxidant enzymes like superoxide dismutase (SOD), catalase (CAT), reduced glutathione (GSH) and glutathione peroxidase (GSHPx). MDA formation was estimated by the method of Slater and Sawyer.[19] Reduced glutathione was determined by the method of Moron *et al*.[20] Superoxide dismutase was determined by the method of Mishra and Fridovich.[21] Catalase was estimated by the method of Hugo Aebi as given by Colowick *et al*.[22] Glutathione peroxidase was determined by the method of Paglia and Valentine.[23]

### Determination of xanthine oxidase activity

Tissue xanthine oxidase (XO) activity was measured spectrophotometrically by the formation of uric acid from xanthine through the increase in absorbance at 293 nm.[24] The phosphate buffer (pH, 7.5) and xanthine were mixed with supernatant sample and then incubated for 30 minutes at 37°C. The reaction was stopped at 0 and 30 minutes by addition of 100% trichloroacetic acid. Thereafter, the mixture was centrifuged at 5000 g for 30 minutes. The activity was measured at 293 nm. One unit of activity was defined as 1 mmol of uric acid formed per minute at 37°C, pH 7.5.

### Determination of nitric oxide level

Nitric oxide (NO) easily breaks down in the presence of free radicals, hence nitrite levels were measured as a level of NO inactivated due to superoxide radical. Nitrite was estimated colorimetrically with the Griess reagent[25] in homogenate at 540 nm. Nitrite was determined from the standard curve obtained using sodium nitrite as standard. The resulting equation was used to calculate the unknown sample concentrations.

### Determination of myeloperoxidase activity

Myeloperoxidase (MPO) activity was determined using a 4-aminoantipyrine/phenol solution as the substrate for MPO-mediated oxidation by H_2_O_2_, and changes in absorbance at 510 nm were recorded.[26] One unit of MPO activity is defined as that which degrades 1 *µ*mol H_2_O_2_ per minute at 25°C.

### Determination of serum creatinine phosphokinase activity

Creatinine phosphokinase (CPK) activity was measured by using a commercial kit (CK-NAC activated, Boehringer Mannheim).

### Statistical analysis

All the values are expressed as mean ± SEM. Statistical significance between more than two groups was tested using one-way ANOVA followed by the Bonferroni multiple comparisons test using a computer-based fitting program (Prism, Graphpad 5.). Differences were considered to be statistically significant when *P* was less than 0.05.

## RESULTS

### Effect of exenatide on liver function

**Figure 2:**
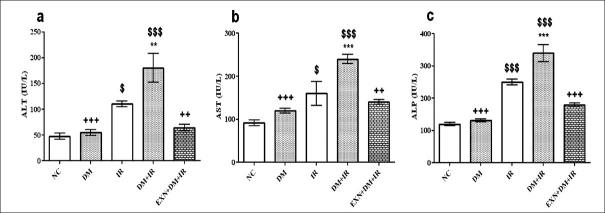
Effect of exenatide on liver function after renal IR in diabetic rats (a: alanine aminotransferase, b: aspartate aminotransferase, c: alkaline phosphatase). Values are mean ± SEM (n= 6), analyzed by one-way ANOVA followed by Bonferroni multiple comparisons test. ^$^, ^*^,+ *P* < 0.05; $$,^**^,++ *P* < 0.01; $$$,^***^,+++ *P* < 0.001. ^$^ Compared with normal control, ^*^ Compared with IR, + Compared with DM+IR group.

Diabetic rats that underwent renal IR exhibited significant increase in the serum concentrations of ALT, AST and ALP as compared to nondiabetic rats (*P* < 0.001), suggesting a significant degree of liver dysfunction caused by renal IR in diabetes. Serum concentrations of ALT, AST and ALP, which were used as markers of liver injury, were significantly reduced subsequent to pretreatment with exenatide prior to IR in diabetic rats (*P* < 0.01) [Figures 2a–2c].

### Effect of exenatide on lipid peroxidation and antioxidant enzymes

**Figure 3:**
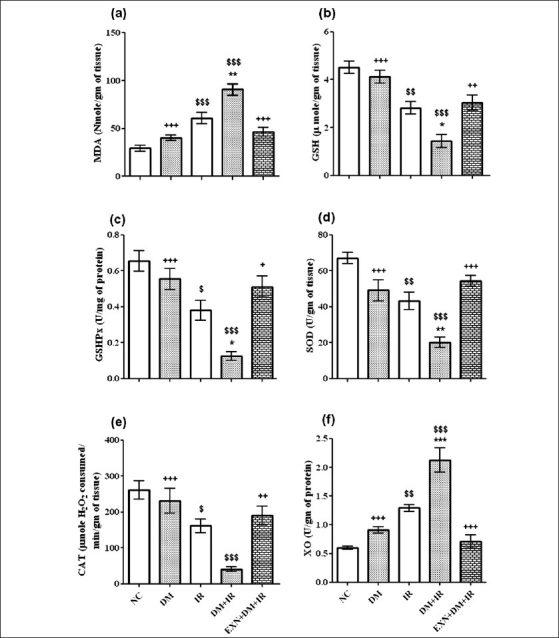
Effect of exenatide on lipid peroxidation (a), reduced glutathione (b), glutathione peroxidase (c), superoxide dismutase (d), catalase (e) and xanthine oxidase (f) in liver after renal IR in diabetic rats. Values are mean ± SEM (n= 6), analyzed by one-way ANOVA followed by Bonferroni multiple comparisons test. ^$^, ^*^,+ *P* < 0.05; $$,^**^,++ *P* < 0.01; $$$,^***^,+++ *P* < 0.001. ^$^ Compared with normal control, ^*^ Compared with IR, + Compared with DM+IR group

The MDA level was significantly increased in nondiabetic IR and in diabetic IR groups compared to normal control (*P* < 0.001) [Figure 3a]; and significant decrease was found in the level of GSH as well as in the activity of SOD (*P* < 0.01) [Figures 3b and 3d] in the nondiabetic IR group as compared to control group. The MDA level was significantly higher in diabetic IR group compared to nondiabetic IR group (*P* < 0.001) [Figure 3a]. Diabetic group demonstrated a significant decrease in GSHPx activity after IR as compared to the control group, and significant difference was observed between nondiabetic IR rats and diabetic IR rats (*P* < 0.05) [Figure 2c]. The CAT activity was significantly lower in diabetic IR group compared to diabetic group (*P* < 0.001) [Figure 3e]. Tissue levels of GSH and SOD were significantly (*P* < 0.05) reduced in diabetic IR group compared to nondiabetic IR group [Figures 3b and 3d]. However, exenatide treatment resulted in a significant decrease in MDA level compared to the diabetic IR group (*P* < 0.001). A significant restoration was observed in the level of GSH, GSHPx, SOD and CAT in exenatide pretreatment group as compared to diabetic IR group [Figures 3b–3e].

### Effect of exenatide on xanthine oxidase activity

The XO enzyme activity, one of the sources of ROS production, was significantly increased (*P* < 0.001) in diabetic IR group compared to normal control group. In the diabetic IR group, XO activity significantly increased (*P* < 0.001) compared to nondiabetic IR rats. In contrast, the exenatide treatment group showed significantly decreased (*P* < 0.001) in XO as compared to diabetic IR rats [Figure 3f].

### Effect of exenatide on nitric oxide level

**Figure 4:**
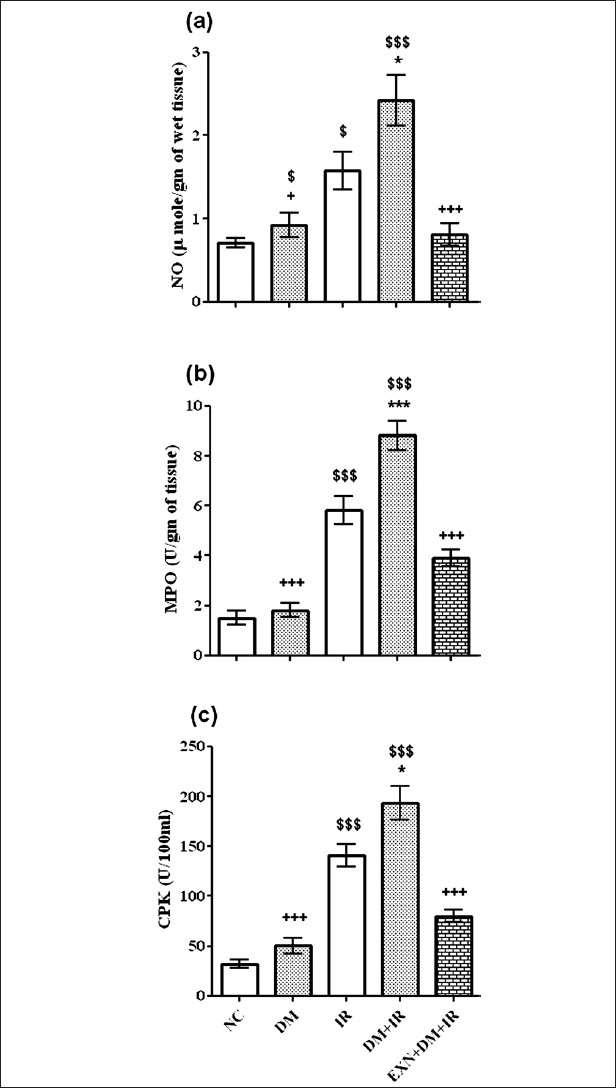
Effect of exenatide on nitric oxide (a), myeloperoxidase (b) and serum creatinine phosphokinase activity (c) after renal IR in diabetic rats. Values are mean ± SEM (n= 6), analyzed by one-way ANOVA followed by Bonferroni multiple comparisons test. ^$^, ^*^,+ *P* < 0.05; $$,^**^,++ *P* < 0.01; $$$,^***^,+++ *P* < 0.001. $Compared with normal control, ^*^ Compared with IR, + Compared with DM+IR group

The level of NO was significantly increased in nondiabetic IR and diabetic IR groups in comparison with normal control group (*P* < 0.05, *P* < 0.001) respectively. Diabetic IR group had significantly (*P* < 0.05) high NO level as compared to nondiabetic IR group. Exenatide pretreatment group showed significantly decreased NO levels in comparison with diabetic IR rats (*P* < 0.01) [Figure 4a].

### Effect of exenatide on myeloperoxidase activity

Myeloperoxidase activity, which is accepted to be an indicator of neutrophil infiltration, was significantly higher in the liver tissue of the diabetic IR group than in the liver tissue of the nondiabetic IR group (*P* < 0.001). Rats in the exenatide pretreatment group had significantly lower MPO activity than the rats in the diabetic IR group (*P* < 0.001) [Figure 4b].

### Effect of exenatide on creatinine phosphokinase activity

Nondiabetic and diabetic rats demonstrated a a significant increase (*P* < 0.001), in CPK activity after IR. In the diabetic IR group, CPK activity was found to be significantly increase (*P* < 0.001), in comparison with nondiabetic IR rats. In contrast, exenatide treatment group showed significantly increase (*P* < 0.01), in CPK as compared to diabetic IR rats [Figure 4c].

## DISCUSSION

In the present study, ALT, AST and ALP activities did not increase after renal IR as much as a liver failure. However, their were statistically significant rise in the liver function enzymes after renal IR in diabetic rats as compared to nondiabetic rats, which indicated diminished liver function in diabetes as compared to normal state, which might be attributed to diabetes-potentate liver injury induced by renal IR. Exenatide treatment prevented alterations in ALT, AST and ALP enzyme activities. The present results indicated that renal IR caused distant-organ injury such as in the liver; it was exaggerated by diabetes, and exenatide treatment had a protective role against liver injury induced by renal IR.

We found significantly higher MDA levels in the liver tissue of both nondiabetic and diabetic rats after induction of renal IR injury, which is a major index of lipid peroxidation and oxidative stress. This might be due to ROS production via inflammatory response; as inflammatory reactions are activated during the process of IR injury, resulting in the formation of inflammatory cytokines, like tumor necrosis factor-alpha (TNF-α), interleukin-1 (IL-1) and arachidonic acid metabolites.[27] Cyclooxygenase (COX)-2 is induced in response to pro-inflammatory cytokines, and it catalyzes the metabolism of arachidonic acid. It is reported that 3 to 5 hours after IR injury, COX-2 expression was most intense; and 12 to 24 hours after IR injury, maximal tissue damage was observed. Hence we decided to analyze tissue injury after 30 minutes of ischemia and 24 hours of reperfusion.[27] Demonstration of lipid peroxidation helps to explain better the exact mechanism of effect of renal IR on liver tissue, and it was found significantly higher in our study, indicating generation of oxidative stress.[28]

The cardiac MPO activity increased after renal IR, consistently with leukocyte infiltration and activation. The active neutrophils show high MPO activity in the tissue as an inflammatory response.[29] The present work showed that liver MPO activity was higher in nondiabetic IR group and further increased in diabetic IR group, both of which were similar to those of cardiac results. The finding that liver MPO activity was increased after induction of IR is very important because it clearly shows high leukocyte function in the liver tissue. Neutrophils play a major role in oxidant injury via mechanisms such as the action of nicotinamide adenine dinucleotide phosphate (NADPH) oxidase or MPO system. Hypochlorous acid is produced largely from stimulated neutrophils by MPO activity. Hypochlorous acid causes oxidation of other molecules such as proteins, amino acids, carbohydrates, nucleic acids and lipids, expanding liver tissue damage.[30] In the present study, MPO activity was inhibited by exenatide treatment. This might also result in reduced lipid peroxidation and thus less accumulation of MDA, because activation of neutrophils generates more oxygen-reactive metabolites.

Another radical producing mechanism might be NO-producing system. The reaction of NO with O_2_^· –^ results in peroxynitrite formation, a potent and aggressive cellular oxidant, and causes the formation of 3-nitro-L-tyrosine.[31] Nitrite/ nitrate levels, as the end products of nitric oxide conversion, were increased in blood plasma and aortic tissue in diabetic animals in comparison with nondiabetic animals,[32] which was confirmed by elevated NO levels in our study. Exenatide treatment in diabetic IR animals normalized the elevated tissue NO level, which might be attributed to inhibition of inducible nitric oxide synthase (iNOS) enzyme. Streptozotocin-induced diabetes caused increase in activity and expression of liver iNOS.[33] NO levels were found to be significantly elevated in diabetic liver tissue at a very early stage in the investigation by Stadler *et al*.[34] Exenatide’s reported anti-diabetic effect may decrease activity and expression of iNOS in liver tissue. The present results have demonstrated the involvement of iNOS in the inflammatory process, and it might have a role in distant-organ injury induced by renal IR via activated iNOS-producing cells.

We found higher NO levels in diabetic IR rats in comparison with nondiabetic IR rats, which is the same as reported previously.[35] Liver tissue from the diabetic group did not show any evidence of the occurrence of ROS[35] and is consistent with our findings. Increased NO production in DM did not alter cellular function in liver tissue. Also, DM did not affect the serum liver enzymes ALT and AST, which is in contrast with the finding in the control group. Renal IR injury in STZ–induced diabetes in rats demonstrated increases in lipid peroxidation and nitrite levels,[36] which was confirmed in our study.

The results of the present work demonstrated, exenatide treatment caused decrease in lipid peroxidation in liver tissue after renal IR. Antioxidant enzymes like GSH, GSHPx, CAT and SOD were increased in liver tissue after exenatide treatment, followed by renal damage in diabetic rats. Also exenatide lowered elevated MPO activity. Thus, exenatide protected against liver injury by preventing neutrophil activation and ROS production, as well as by decrease in XO activity. Several mechanisms might be responsible for the protective effects of exenatide against distant-organ injury induced by renal IR in diabetes; one of them, exenatide might reduce apoptosis and oxidative stress, as shown in our study. Exenatide is GLP-1 receptor agonist, and GLP-1 has been reported to protect the rat heart from IR injury, mediated by the pro-survival kinases PI3K/Akt, p42/44 PKA, and P70s6; in that, exenatide treatment caused increase in myocardial expression of pAkt, and expression of active caspase 3 was reduced. Exenatide-treated animals had higher activity of the antioxidant enzymes superoxide dismutase and catalase. Exenatide-treated animals had reduced nuclear oxidative stress.[15–17] Modification in oxidative stress and inflammatory response by exenatide treatment could be the one of the reason of liver-protective effect.

## CONCLUSION

In conclusion, diabetes exaggerated liver damage induced by renal IR via oxidative stress and inflammatory process in STZ-NAD–induced diabetes in rats. Also, the exenatide treatment attenuated liver injury induced by renal IR in diabetic rats. This is the first study in which exenatide was used to prevent liver injury induced by IR in diabetes, the prevention being shown to achieve via NO generation and neutrophil sequestration in the liver tissue.
